# Miscellaneous

**Published:** 1874-04

**Authors:** 


### MISCELLANEOUS.

### A NEW VERSION OF OLD MOTHER HUBBARD.

The widowed dame of Hubbard's ancient line
Turned to her cupboard, cornered anglewise
Betwixt this wall and that, in quest of aught
To satisfy the cravings of Sir Tray,
Pricked-ear companion of her solitude,
Red spotted, dirty white and bare of rib,
Who followed at her high and pattering heels,
Prayer in his eye, prayer in his sinking gait,
Prayer in his pendulous pulsating tail.
Wide on its creaking jaws revolved the door,
The cupboard yawned, deep throated, thinly set
For teeth with antiquent bottles, canisters.
Deep in the void she thrust her hookèd nose,
Peering near sighted for the wished for bone,
While her short robe of samite, tilted high,
The thrifty darnings of her hose revealed—
The pointed feature travelled o'er the deft,
Greasing its tip, but bone or bread found none,
Wherefore Sir Tray abode still dinnerless,
Licking his paws beneath the spinning wheel,
And meditating much on savory meats.

### RECONCILIATION.

Entire reconciliation is difficult with a woman. She invariably keeps certain reserves. When she has once parted from you in spirit she will hardly return. Though she seems to she does not. She gives her hand again—perhaps her lips; but the heart is no longer in one nor the soul in the other. Kiss her you have once roundly quarrelled with—if it be not a mere lovers' quarrel—and you will find the statue under the crimson curve, the chill of the marble through the bounding blood. A keen observer may determine in society whether you have had discord with the women you meet. However perfect the breeding, however disciplined the manners, the past discord leaves a shadow that will not be lifted. The old wound may be closed, it is not healed, nor can it be by the highest skill in spiritual surgery. Frequently men like one another better after fighting; women never, be the foe of either sex. With these the bloom of favor is taken off, not to be restored. They feel, though they may not say or even think it, that slight or injury admits of no atonement. Woman reads the proverb: To err is feminine, to forgive impossible.—*The Galaxy.*

### JAPANESE WOMEN.

NATURALLY there are no figures more perfect than those of the Japanese young women. The children, up to the age of fourteen, or as long as they have the free use of their limbs, are models of symmetry. About that time they begin to fasten long garments about their hips, the effect of which is to impede their gait and give them an awkward shamble. In course of time it does worse, and interrupts the development of the legs and thighs. Among the laboring class an additional misshapening is accomplished by the practice of carrying burdens from an early age upon the back, for the support of which broad straps are passed over the shoulders and crossed in front, pressing directly upon the breasts. When a Japanese girl reaches the age of sixteen without having undergone either of the processes of deformity, she is a wonder to the eye, and remains so until twenty-five, or possibly a little later; then she ceases to charm for a certain period, in any way excepting by her manner, and that is generally preserved to the last. But as she grows old she has a chance of becoming quite delightful again. There is nothing nicer than a dignified and white haired old Japanese lady. She is always happy, for she is always much respected and cherished by her youngers, and at a certain age the natural high breeding of the race appears in her to attain its crystallizaton. Whatever her station in life, she is almost always sure to suggest an idea of ancient nobility, and to be surrounded by an atmosphere of an Oriental Faubourg St. Germain.—*Atlantic Monthly.*

### A NATIONAL WATCHWORD.

Mrs. Admiral Dahlgren writes: "If Peru has a watchword it must be 'manana' (to-morrow); and in this disposition to delay until to-morrow what may just as well be done to-day may, perhaps, be found one reason why the country remains in so many respects retrograde. One fair morning the United States consul, who is one of the most amiable of men, makes us a call. He lives in Callao, and only comes up to Lima now and then. On this occasion he has such a wearied, disappointed look, that we involuntarily ask, 'What is the matter?' 'The matter? Why, "manana."' 'What do you mean?' we interrogate. 'Well, I mean that nothing can be accomplished here definitely; you are always politely met, patiently listened to, and answered "manana." I am sick of it. For six months past I have been coming up here at intervals, by appointment of State officials, to attend to a matter which ought to be adjusted in one half hour. When, for the thousandth time to-day, I thought to have reached the end, I am bowed out with "manana." For once I lose my temper, and exclaim: 'But, your excellency, it is "manana" forever, and I suppose in the other world it will be the same, "manana."' We laugh, but we, too, hear it every day. In all one's business affairs delay, never ending delay. The enervating climate, doubtless, has something to do with this, but it is also in a measure to be attributed to the characteristic subtlety of this people. They are wary, especially of strangers, and even though distrustful, are too kind hearted and mild tempered expressly to dissent. Hence the compromise of 'manana.'"

### A STORY OF THE CURFEW.

The first line of Gray's Elegy,

"The curfew tolls the knell of parting day,"

has made the word *curfew* familiar to every English speaking boy and girl. The word is formed of two French words, *couvre feu* or *couvrir feu* (cover fire), and came into use when William the Norman, the first monarch of England, made a law that all fires should be extinguished at the sound of the evening bell.

To many hearts in the old country that cherish its traditions, the "curfew" recalls a story of love's devotion:

In the time of Cromwell a young soldier, for some offence, was condemned to die, and the time of his death was fixed "at the ringing of the curfew." Naturally such a doom would be fearful and bitter to one in the years of his hope and prime, but to this unhappy youth death was doubly terrible, since he was soon to have been married to a beautiful young lady whom he had long loved.

The lady, who loved him ardently in return, had used her utmost efforts to avert his fate, pleading with the judges and even with Cromwell himself, but all in vain. In her despair she tried to bribe the old sexton not to ring the bell, but she found that impossible. The hour drew near for the execution. The preparations were completed. The officers of the law brought forth the prisoner, and waited, while the sun was setting, for the signal from the distant bell tower.

To the wonder of everybody it did not ring! Only one human being at that moment knew the reason. The poor girl, half wild with the thought of her lover's peril, had rushed unseen up the winding stairs, and climbed the ladders into the belfry loft, and seized the tongue of the bell.

The old sexton was in his place, prompt to the fatal moment. He threw his weight upon the rope, and the bell, obedient to his practiced hand, reeled and swung to and fro in the tower, but the brave girl kept her hold, and no sound issued from its metallic lips.

Again and again the sexton drew the rope, but with desperate strength the young heroine held on. Every movement made her position more fearful, every sway of the bell's huge weight threatened to fling her through the high tower window, but she would not let go.

At last the sexton went away. Old and deaf, he had not noticed that the curfew gave no peal, the brave girl descended from the belfry, wounded and trembling. She hurried from the church to the place of execution. Cromwell himself was there, and was just sending to demand why the bell was silent. She saw him—

——" And her brow,
Lately white with sickening horror, glows with hope and courage now;
At his feet she told her story, showed her hands all bruised and torn,
And her sweet young face, still haggard with the anguish it had worn,
Touched his heart with sudden pity, lit his eyes with misty light—
'Go ! your lover lives,' cried Cromwell ; 'curfew shall not ring to-night.' "

—*Youth's Companion.*

### PRAYER OF COLUMBUS.

[It was near the close of his indomitable and pious life—on his last voyage, when nearly 70 years of age—that Columbus, to save his two remaining ships from foundering in the Caribbean Sea in a terrible storm, had to run them ashore on the Island of Jamaica—where, laid up for a long and miserable year—1503—he was taken very sick, had several relapses, his men revolted, and death seemed daily imminent; though he was eventually rescued and sent home to Spain to die, unrecognized, neglected, and in want. * * * * It is only asked, as preparation and atmosphere for the following lines, that the bare, authentic facts be recalled and realized, and nothing contributed by the fancy. See the Antillean island, with its florid skies and rich foliage and scenery, the waves beating the solitary sands, and the hulls of the ships in the distance. See the figure of the great admiral walking the beach, as a stage, in this sublimest tragedy—for what tragedy, what poem so piteous and majestic as the real scene? and hear him uttering—as his mystical and religious soul surely uttered—the ideas following, perhaps, in their equivalents, the very words.]

A batter'd, wreck'd old man,
Thrown on this savage shore, far, far from home,
Pent by the sea, and dark rebellious brows, twelve dreary months;

Sore, stiff with many toils, sicken'd and nigh to death,
I take my way along the island's edge,
Venting a heavy heart.

I am too full of woe !
Haply I may not live another day;
I cannot rest, O God—I cannot eat, or drink, or sleep,
Till I put forth myself, my prayer, once more to Thee—
Breathe, bathe myself once more in Thee—commune with Thee,
Report myself once more to Thee.

Thou knowest my years entire, my life ;
(My long and crowded life of active work—not adoration merely;)
Thou knowest the prayers and vigils of my youth;
Thou knowest my manhood's solemn and visionary meditations;
Thou knowest how, before I commenced, I devoted all to come to
Thee;
Thou knowest I have in age ratified all those vows and strictly
kept them;
Thou knowest I have not once lost nor faith nor ecstacy in Thee ;
(In shackles, prison'd, in disgrace, repining not,
Accepting all from Thee—as duly come from Thee).

All my emprises have been fill'd with Thee,
My speculations, plans, began and carried on in thoughts of Thee,
Sailing the deep or journeying the land for Thee;
Intentions, purports, aspirations mine—leaving results to Thee.

O I am sure they really came from Thee—
The urge, the ardor, the unconquerable will,
The potent, felt, interior command, stronger than words,
A message from the heavens, whispering to me even in sleep—
These sped me on.

By me and these, the work so far accomplish'd (for what has been
has been);
By me Earth's elder, cloy'd and stifled lands, uncloy'd, unloos'd,
By me the hemispheres rounded and tied—the unknown to the
known.

The end I know not—it is all in Thee;
Or small or great I know not, haply, what broad fields, what
lands;

Haply the brutish, measureless, human undergrowth I know,
Transplanted there, may rise to stature, knowledge worthy Thee;
Haply the swords I know may there be turn'd to reaping tools;
Haply the lifeless cross I know—Europe's dead cross—may bud
and blossom there.

One effort more—my altar this bleak sand;
That thou, O God, my life hast lighted
With ray of light, steady, ineffable, vouchsafed of Thee,
(Light rare, untellable—lighting the very light!
Beyond all signs, descriptions, languages!)
For that, O God—be it my latest word—here on my knees,
Old, poor and paralyzed—I thank Thee!

My terminus near,
The clouds already closing in upon me,
The voyage balk'd—the course disputed, lost,
I yield my ships to Thee.

Steersman unseen! henceforth the helms are thine;
Take Thou command—what to my petty skill Thy navigation?

My hands, my limbs grow nerveless;
My brain feels rack'd, bewildered;
Let the old timbers part—I will not part!
I will cling fast to Thee, O God, though the waves buffet me;
Thee, Thee, at least, I know.

Is it the prophet's thought I speak or am I raving?
What do I know of life—what of myself?
I know not even my own work, past or present;
Dim, ever shifting guesses of it spread before me—
Of newer, better worlds—their mighty parturition
Mocking, perplexing me.

And these things I see suddenly—what mean they—
As if some miracle, some hand divine unsealed my eyes?
Shadowy, vast shapes, smile through the air and sky,
And on the distant waves sail countless ships,
And anthems in new tongues saluting me.

*Walt. Whitman.*

### BEECHER ON THEOLOGIANS.

Henry Ward Beecher, in a recent lecture to the students of Yale Theological School, said: "I believe in theologians, and yet I think it is perfectly fair to make game of them! I do not think there is anything in this world, whether it be man or that which is beneath man, that is not legitimate food for innocent, unvicious fun; and if it should cast a ray of light on the truth, and alleviate the tediousness of a lecture now and then to have a slant at theologians, why I think they can stand it! It will not hurt them and it may amuse us."

### ANECDOTE OF WEBSTER.

When Daniel Webster had reached the very topmost height of his fame, after his great speech in reply to Colonel Hayne, of South Carolina—the speech commonly known as his "Constitutional Speech"—he paid a visit to his old home in New Hampshire, the neighborhood of his boyish years and his first manly aspirations and strugglings. A well known citizen was his companion. After going up the rough mountain road for a good long while Webster pointed out the nearness of the old parental roof-tree. "There," said he, "is neighbor"—Jones, we will call him—"there's our old neighbor, Jones. I'll stop and talk with him and see if he knows me." So Mr. Webster got out of the wagon and walked on ahead. Soon he met the old man Jones and "passed the time of day," as they say in good, rural New England. Webster walked lazily, loiteringly along the road, and, finally turning, said to the seamed, gnarled, rugged old farmer, "Wasn't there a family named Webster once living near you? I knew something of a family of that name said to live in these parts."

"Why, yes," said Jones. "Webster? yes, our old neighbor. He had two likely boys. Les-see; Zeke, and then there was—what's his name—O, Dan'l. Dan'l Webster." And then Daniel, leaning on the fence, engaged in a long talk with the farmer about the Webster family—a talk quite unnecessary to reproduce here. The farmer was very enthusiastic about Ezekiel. Ezekiel

Webster, it should be remembered, was a young man of rare promise, of even greater promise than his famous brother, as we believe the latter admitted in riper years. He died when but a young man. The farmer could not say too much in praise of Ezekiel—to all of which, of course, Daniel Webster listened with boundless satisfaction. But finally the latter said, "What became of the other brother—Daniel?"

"Oh, I don't know," said farmer Jones. "He went away, and I believe is a kind of lawyer down in Boston."—*Golden Age.*

### A LOBSTER AT DINNER.

A LOBSTER is a particular fellow in his food. If a portion of food be thrown down to him he immediately sets his long horns at work to ascertain the whereabouts of his dinner. If he does not like it he at once pushes it away from him with the attitude of an epicure who bids the waiter take away a plate of meat he does not fancy. If the food is agreeable to him he munches it up, moving his jaws in a peculiar way, like a weaver making a blanket. He tears his food into large pieces, leaving the actual pounding work to be done by the very peculiar internal teeth, which are found in the lining of the stomach, and which the reader can easily examine for himself, if he will take the trouble. When the lobster goes out for a "constitutional," and is not in a particular hurry, he carries his great claws in front of him, well away from the ground, like the big flags we sometimes see heading street processions. He "walks" upon the little legs which are underneath his body, while he keeps his horns moving in front of his nose like a blind man tapping the flags with his stick as he plods along, led by his dog. If the least thing alarms him he scuttles backward on his little legs, which move with the rapidity of the legs of the centipede. If he does not go fast enough in this way he suddenly snaps his tail toward him, like a man suddenly closing his hand, and flies backward with a jerk like an India rubber band snapped in half. He always goes into his cave tail foremost, and he takes the most wonderfully good shots at the entrance. It has been said that a fly fisherman will never be perfect until he has got an eye at the back of his head, so as to prevent his drop fly getting hitched up in the tree behind him. The lobster must have an eye in his tail somewhere. The lobster is not willing that the secrets of her toilet should be exposed to vulgar gaze, so she artfully collects cockle and oyster shells and makes a trench round herself, after the fashion of the Romans when they took possession of a hill-top. A branch of sea-weed forms a canopy over her head, and there she is in a house of her own making—a regular "compound householder," with no taxes to pay.

### A BIT OF NATURE IN THE PARLOR.

WHILE in Jacksonville, Fla., we saw something so pretty, and in such taste, that we will try to describe it, and perhaps some of our northern ladies may avail themselves of the idea, which may be developed in many ways. In a shadowy corner of the room a shelf was covered with a large quantity of various kinds of dried native grasses, over which the Spanish moss of Florida was hanging in festoons from short branches until it almost touched the tops or mingled with the grasses. Within it looked as dreamy and as dark as the recesses of the swamps and forests. Just among the grasses, and half concealed by the hanging moss, stood a beautiful, small, snow-white heron, nicely and perfectly stuffed, and looking remarkably life-like. He seemed to be standing in his native marsh, and his attitude was as if he had just spied the observer, and, startled by his intrusion, was about to take flight. The poise of the head and the wild glance of the eye were perfect. The illusion was complete, and the whole formed one of the most tasteful and beautiful decorations for a parlor we have ever seen.—*Forest and Stream.*

### SPARROWS AND WORMS.

THE rural populations of France, a few years ago, were strikingly reminded of the necessity of preserving the birds, and the Legislature was overrun with petitions for a law to that effect. The number of birds had been diminished from year to year while that of injurious insects were correspondingly increased. It was estimated by those who at the time took upon themselves the careful investigation of the subject, that more than one hundred millions of birds' eggs were destroyed annually in the country. How many millions of insects this wholesale wantonness preserved in injurious existence it would be difficult to say, but some idea of the number may be formed from the fact that in the stomach of an insectivorous bird, killed as it was entering its nest for the night, was found a thousand insects of different species.

The ravages of the grasshopper have been so enormously destructive during the past year at the West, particularly in Iowa and Minnesota, as to impoverish large numbers of farmers through the entire loss of their crops, upon which they exclusively depended for subsistence. It may be that no description or number of insectivorous birds could have prevented or resisted such a terrible raid as this ; but it is believed that the districts of country which are most infected by injurious insects are those where the presence of birds is the least encouraged, or rather where there is the largest indulgence in the manly sport of destroying them.

The English sparrow, which, from its fecundity as well as its voracity, is one of the most efficient insect destroyers, has been imported into this country to some extent, but it has never been so encouraged and protected as to enjoy a fair opportunity to multiply its forces and to exhibit its insectivorous power. An intelligent cotton planter at the South expresses an opinion, as the result of his own observation, that the presence of the English sparrow in the cotton belt would effectually wipe out the cotton worm, the ravages of which are estimated by tens of millions of dollars. In Germany the laws against the destruction of the sparrow are so stringent that there is a constant surplus of the birds for exportation, and they are carried in large numbers to distant points. There is a public botanical garden at Melbourne, in Australia, which is described as being a superb one. It is the little kingdom of Baron Von Mueller, who is well known in this country as a zealous and scientific naturalist. He is the most liberal of sovereigns, for every day he gives liberty to hundreds of his subjects. These are simply sparrows, which are sent to him from Germany in cages of three hundred each. Every German vessel that anchors at Port Philip carries thousands of these little but energetic birds, to be preserved and utilized in destroying the injurious insects of Australia.—*Boston Traveller.*

### MEMORY OF FACES.

WHEN a man has the painter's faculty of recollecting faces, and with it a quick and retentive memory of small facts, the combination gives him great social power. This was Macaulay's case. He never forgot the face of a man whom he had met in society, and with the face he remembered all the salient facts connected with the owner of it. Few things are more flattering to an ordinary mortal than being thoroughly remembered by a great lion with whom he has perhaps had a brief interview several years before. I doubt if this faculty exists to any great extent among our public men, indeed I have often been surprised at the absence of it. A Russian baron, of the true divine right school, once maintained to me that this was an effect of republican institutions, or, what came to the same thing, that the opposite was the result of monarchial institutions. He said that kings and princes were obliged to see a great many persons, wherefore Providence had conferred on them various means of being gracious to those persons, of which prompt recognition was one.—*Galaxy.*

### CAN THE ETHIOPIAN CHANGE HIS SKIN?

DR. G. T. MAXWELL, of New Castle, Pa., details an interesting experiment which he tried, and which adds peculiar force to the question asked by the prophet Jeremiah. In 1872 a negro named James Pearce was shot in the face accidentally, the wound being such as would leave a frightful scar unless disfigurement could be in a measure prevented by the engrafting of new skin upon the mutilated portion of the face. This course was adopted, and with his patient's consent Dr. Maxwell took grafts of skin from his own arm, which, with other grafts from the patient's arm, he placed upon the surface of the wound. The success of the operation was seriously endangered by the patient going on a spree, during which he destroyed some of the grafts, but fortunately two were left, one of them being white. The piece of white skin, which at first was only the size of a canary seed, grew to cover a space of half an inch, and for a time after the wound had healed could be easily discerned at quite a distance. Close examination at this time showed that dark colored lines formed a network in the skin, giving a purplish tinge to it. At the end of a month these lines, which were simply blood vessels, had increased to such an extent that the whole surface of the wound was of uniform hue, the white skin having lost all its characteristics. From this experiment Dr. Maxwell draws the conclusion that the coloring matter which darkens the skin of some races is in the blood, and not in the skin itself.

### HOME INFLUENCE.

Women who have sons to rear, and dread the demoralizing influences of bad associates, ought to understand the nature of young manhood. It is excessively restless. It is disturbed by vague ambitions, by thirst for action, by longings for excitement, by irrepressible desires to touch life in manifold ways. If you, mothers, rear your sons so that their homes are associated with the repression of these natural instincts, you will be sure to throw them into the society that in any measure can supply the need of their hearts. They will not go to public-houses, at first, for love of liquor—very few people really like the taste of liquor—they will go for the animated and hilarious companionship they find there, which, they discover, does so much to repress the disturbing restlessness in their breasts. See to it, then, that their homes compete with public places in attractiveness. Open your blinds by day and light bright fires at night. Illuminate your rooms. Hang pictures upon the walls. Put books and newspapers upon your tables. Have music and entertaining games. Banish those demons of dulness and apathy that have so long ruled in your household, and bring in mirth and good cheer. Invent occupations for your sons. Stimulate their ambitions in worthy directions. While you make home their delight, fill them with higher purposes than mere pleasure. Whether they shall pass happy boyhoods, and enter upon manhood with refined tastes and noble ambitions, depends upon you. Do not blame miserable barkeepers if your sons miscarry. Believe it possible that, with exertion and right means, a mother may have more control over the destiny of her boys than any other influence whatsoever.—*Appleton's Journal.*

### THE VILLE DU HAVRE.

BY MARY F. VAN DYCK.

Serenely sleeping, in the dead of night,
They heard no noise of Azrael's sable wing—
From Zion's watch tower saw no beacon light,
Or snowy hand of angel beckoning.

At many hearthstones stands a vacant chair,
In many heads the fount of tears is dry;
Oh! pitying Heaven, send balm of Gilead there :
Blest are the eyes that see their loved ones die.

Had we but clasped them in a last embrace,
And kissed the lips that oft had sought our own,
Then laid them in some sweet sequestered place,
Afar from ocean's boasting monotone,

With patience we would wait the promised time
When flaming tongues shall lick the ocean dry,
When spirits gather in their natal clime,
Where lips ne'er utter, "We are born to die!"

Our bodies are but dust that falls in sight,
When the enfranchised spirit soars on high,
But living soul and body reunite,
And life eternal crowns the victory.

